# High Female Survival Promotes Evolution of Protogyny and Sexual Conflict

**DOI:** 10.1371/journal.pone.0118354

**Published:** 2015-03-16

**Authors:** Tobias Degen, Thomas Hovestadt, Oliver Mitesser, Franz Hölker

**Affiliations:** 1 Leibniz-Institute of Freshwater Ecology and Inland Fisheries, Berlin, Germany; 2 Institute of Biology, Freie Universität Berlin, Berlin, Germany; 3 Field Station Fabrikschleichach, Department of Animal Ecology and Tropical Biology, Biozentrum, Universität Würzburg, Rauhenebrach, Germany; 4 Department of Biology, Terrestrial Ecology Unit, Ghent University, Ghent, Belgium; University of Thessaly, GREECE

## Abstract

Existing models explaining the evolution of sexual dimorphism in the timing of emergence (SDT) in Lepidoptera assume equal mortality rates for males and females. The limiting assumption of equal mortality rates has the consequence that these models are only able to explain the evolution of emergence of males before females, i.e. protandry—the more common temporal sequence of emergence in Lepidoptera. The models fail, however, in providing adaptive explanations for the evolution of protogyny, where females emerge before males, but protogyny is not rare in insects. The assumption of equal mortality rates seems too restrictive for many insects, such as butterflies. To investigate the influence of unequal mortality rates on the evolution of SDT, we present a generalised version of a previously published model where we relax this assumption. We find that longer life-expectancy of females compared to males can indeed favour the evolution of protogyny as a fitness enhancing strategy. Moreover, the encounter rate between females and males and the sex-ratio are two important factors that also influence the evolution of optimal SDT. If considered independently for females and males the predicted strategies can be shown to be evolutionarily stable (ESS). Under the assumption of equal mortality rates the difference between the females’ and males’ ESS remains typically very small. However, female and male ESS may be quite dissimilar if mortality rates are different. This creates the potential for an ‘evolutionary conflict’ between females and males. Bagworm moths (Lepidoptera: Psychidae) provide an exemplary case where life-history attributes are such that protogyny should indeed be the optimal emergence strategy from the males’ and females’ perspectives: (i) Female longevity is considerably larger than that of males, (ii) encounter rates between females and males are presumably low, and (iii) females mate only once. Protogyny is indeed the general mating strategy found in the bagworm family.

## Introduction

In many annual insects, males and females emerge not exactly at the same time of the season (sexual dimorphism in timing of emergence, SDT) [1]. An analogous observation in long-lived animals such as birds, amphibians, or mammals, is the different timing of arrival to breeding areas by males and females [2]. The more typical observation is that males emerge or arrive earlier than females (“protandry”) [1–4]. In either case, proper timing of activity can have important fitness implications [5]. A specific problem faced by many insects is timing emergence to maximise mating success [6, 7]. It is obvious that the fitness maximizing strategy of members of one sex will critically depend on timing decisions taken by members of the other sex and possibly also of their own sex. Thus, (evolution of) timing provides potential for a sexual conflict over the optimal SDT, i.e. a conflict between the evolutionary interests of individuals of the two sexes [8].

Models providing reasonable explanations for the evolution of protandry in Lepidoptera and other organisms have already been developed (reviews: [2, 9]). Provided that females mate just once but males may mate several times (polygyny), males emerging ahead of females could increase their chance of multiple encounters with virgin females, thus increasing the expected number of matings [3, 10]. Moreover, protandry may reduce the females’ waiting before finding a mating partner [10, 11] and thus, considering female mortality, the risk to die as virgin [10]—an important benefit for a short-lived individual [5]. The model by Zonneveld and Metz [10] showed that the optimal SDT as seen from the perspective of females are almost always slightly different from the SDT maximizing the males’ fitness expectation. Other models which take both perspectives into account also predict only weak sexual conflict over the optimal level of protandry (review: [9]). These models assume genetic control of emergence: “two sex-limited loci: M expressed in males, F in females. […] There will be inter-locus conflict: any mutant allele at M or F allowing cost-free achievement of the optimal outcome for the male or female will spread” [12].

The inverse situation, i.e., that females emerge before males (“protogyny”), occurs far too often among Lepidoptera to accept the lack of an evolutionary explanation [13–22]. Honek [13] reviewed studies of 97 non-diapausing species of insects and found protogyny for complete development in 18% of the species. For the bagworm family (Lepidoptera: Psychidae) that includes approximately 1000 species, protogyny is even the rule [19]. Recent studies have concluded that protogyny—in contrast to protandry—would increase the risk of female mating failures; there should be strong selection to reduce protogyny [1, 23, 24]. Consequently, Rhainds and Gries [24] assumed that there must be benefits other than maximising mating success associated with protogyny, such as avoidance of inbreeding.

As Rhainds [1] already recognised, none of the models mentioned above provides a good explanation for the evolution of protogyny. Because protogyny is wide-spread in bagworms it seems likely that specific life history trait(s) occur in this taxonomic group (in contrast to others), that have not been accounted for in the former modelling approach. Noticeable facts that have been reported for several bagworm species include a high proportion of unmated females, a male-biased sex ratio and neonatal larvae dispersal by ballooning on silken threads [7, 10, 19, 23]. Not considered in any model but specific and wide spread for bagworms is the short lifespan of adults, with a pronounced difference in female and male survival: Male longevity is only one or two days, whereas females may survive for up to two weeks (cf. [19]). In butterflies in general, mortality rates of males are typically only slightly higher than those of females (estimated from wild populations [25, 26]). We refer to studies of wild populations because longevity of sexes can differ significantly between wild and laboratory populations [27, 28]. Nonetheless, that mortality rates differ between genders is known for many other taxa too [25, 26, 29–31].

Thus, the assumption of similar mortality rates underlying previous models may often be violated. Here, we will develop a model that shows that an explanation for the evolution of protogyny may be provided if we consider the effect of uneven mortality rates for females and males. We will show that protogyny may indeed evolve if female mortality is lower than male mortality and that such selection may at the same time intensify the possible sexual conflict over the optimal SDT.

## Materials and Methods

The question we especially want to answer is under which conditions an emergence of females ahead of males may be an adaptive (and evolutionary stable) strategy. Zonneveld and Metz [10] already provided a very useful model to investigate the evolution of SDT; we will present here a straightforward extension of this model by relaxing just one critical assumption. Consequently, we provide only a brief summary description of the model because most details can be found in the paper of Zonneveld and Metz [10]. Our model is based on the following assumptions:

1. The time to emergence of males and females is logistically distributed, with a common scale parameter (*σ*) that is proportional to the standard deviation; only the mean dates of eclosion of males (*μ*_*m*_) and females (*μ*_*f*_) are under genetic control. Generations do not overlap.
2. Death rates of males (*α*_*m*_) and females (*α*_*f*_) are constant but not necessarily equal.
3. The (overall) sex-ratio in the population is the ratio of the total number of females (1 − *ψ*) to the total number of males (*ψ*) emerging over the course of the season. Note that this ratio is typically not identical with the operational sex ratio at a specific moment in time.
4. Females mate at most once or die unmated if they fail to find a mating partner, whereas males are principally capable of multiple matings.
5. Mating occurs as soon as a male encounters a virgin female. The duration of copulation is negligibly small in comparison with the total life expectancy. Consequently, mating does not reduce a male’s chance for future matings.
6. Encounters between females and males occur randomly with rate *γ*.
7. Populations are assumed to be so large that stochastic effects or population extinctions can be ignored.

This list of assumptions is completely identical to that of Zonneveld and Metz [10] with the exception of assumption 2): We analysed the effect of unequal mortality rates in females and males for the selection of SDT. Beyond this, we explored how these effects are modified by encounter rates and uneven sex-ratios.

Reproductive success of a female is critically bound to the fact that she must be fertilised before she dies as a virgin. For males, reproductive success is defined by the number of matings achieved during their lifetime, i.e., the number of virgin females encountered. Given the assumptions above, the temporal dynamics of males (*M̃*) and virgin females (*Ṽ*) is defined by the following set of differential equations:

$$\begin{array}{c} \frac{\partial \tilde{M}\left(t\right)}{\partial t}=\psi \tilde{g}\left(t;\sigma ;\mu_{m}\right)-\alpha_{m}\tilde{M}\left(t\right) \end{array}$$ (1)

and

$$\begin{array}{c} \frac{\partial \tilde{V}\left(t\right)}{\partial t}=\left(1-\psi \right)\tilde{g}\left(t;\sigma ;\mu_{f}\right)-\left(\alpha_{f}+\gamma \tilde{M}\left(t\right)\right)\tilde{V}\left(t\right) \end{array}$$ (2)

Male and virgin female densities increase (the first term in either equation) due to the emergence of new individuals according to a logistic probability density function (*g̃*(*t*; *σ*; *μ*)) with given variance (*σ*) and mean male (*μ*_*m*_) and female (*μ*_*f*_) day of eclosion (parameters and functions are given in Table 1). Male density decreases due to mortality (second term of equation (1)). Similarly, the density of virgin females declines due to mortality—in which case females fail to reproduce—but in addition virgin females are removed from the pool the moment they successfully mate (equation (2)).

**Table 1:** List of parameters and functions used.

parameter	interpretation
*σ*	scale parameter (standard deviation) of log-normal distribution of emergence times
*μ* *	mean of *g*
*α* *	unscaled mortality rate
*ψ*	proportion of males among adults at eclosure
*γ*	female-male encounter rate
*N*	total number of individuals emerging per unit area
*λ* *	scaled mortality rate: *λ* = *αγ*
*φ*	scalede encounter rate: *Nγσ*
*τ*	scaled SDT: *τ* = (*μ*_*f*_ − *μ*_*m*_)/*σ*

function	interpretation
*T*(*t*)	scaled time: *T* = (*t* − *μ*_*f*_)/*σ*
*M*(*T*)	scaled male density at time *T* (scaled to *N*)
*V*(*T*)	scaled density of vergin females at time *T* (scaled to *N*)
*g*(*T*; *μ*)	scaled probability distribution of emergence times
Φ(*T*)	number of females a male emerging at scaled time *T* is expected to mate with
*E*(*τ*)	expected number of matings achieved by males emerging on average *τ* time units
	before females

* with additional subscripts _*m*_ and _*f*_ indicating male respectively female specific parameters.

These equations can be scaled without any loss of generality to simplify the analysis. As male (*α*_*m*_) and female (*α*_*f*_) mortality rates are constant (but not necessarily equal), scaling can be performed analogous to Zonneveld and Metz (cf. [10] page 311ff]). Therefore, we only present the major steps: (1) The individual numbers are scaled by the total number of butterflies (*N*) that emerge per unit of area. (2) The time (*t*) is substituted by the scaled time (*T* = (*t* − *μ*_*f*_)/*σ*) resulting in scaled death rates (*λ*_*i*_ = *α*_*i*_ *σ* (*i* ∈ {*m*, *f*}) and in a scaled encounter rate (*φ* = *Nγσ*). We take the mean female eclosure time as reference time (*T* = 0).

The scaled density dynamics of males (*M*(*T*)) and virgin females (*V*(*T*)) are:

$$\begin{array}{c} \frac{\partial M(T)}{\partial T}=\psi g\left(T;\tau \right)-\lambda_{m}M\left(T\right) \end{array}$$ (3)

and

$$\begin{array}{c} \frac{\partial V(T)}{\partial T}=\left(1-\psi \right)g\left(T;0\right)-\left(\lambda_{f}+\varphi M\left(T\right)\right)V\left(T\right) \end{array}$$ (4)

with the (scaled) logistic probability density

$$\begin{array}{c} g\left(T;\mu \right)=\frac{e^{T+\mu }}{{\left(1+e^{T+\mu }\right)}^{2}} \end{array}$$ (5)

Parameter $\tau =\frac{\mu_{f}-\mu_{m}}{\sigma }$ is the scaled degree of SDT (sSDT). As the mean time of female eclosion is 0 by definition, sSDT (*τ*) simply characterises the relative shift of male eclosion. A positive value of sSDT (*τ*) indicates protandry and a negative one protogyny as in Larsen and Calabrese [23]. Note that the scaled equations are identical to the unscaled equations for *σ* = 1 and *N* = 1. The solution of equations (3) and (4) is identical to the solution of Zonneveld and Metz (cf. [10] page 311ff]) except for the difference in male (*λ*_*m*_) and female (*λ*_*f*_) mortalities (see S1 appendix. for details).

Reproductive success of females with respect to males will depend on the level of SDT in a population—natural selection should thus change the level of SDT so that it maximises success. However, as already explained in the introduction it is important to recognise the different perspectives of females and males in this context: For females the best SDT would be that minimising the risk of dying as unmated virgins; for males the ideal strategy is that maximising the number of matings. Thus, it is not assured that females and males will ‘agree’ on the optimal strategy, i.e., that a joint evolutionarily stable strategy (ESS) exists.

The number of females a male emerging at scaled time *T*_*e*_ is expected to mate with is

$$\begin{array}{c} \Phi \left(T_{e}\right)=\int_{T_{e}}^{\infty }e^{-\lambda_{m}\left(T-T_{e}\right)}\varphi V\left(T\right)dT \end{array}$$ (6)

Equation (6) combines the probability that a male is still alive at a given time (negative exponential term) with the fraction of females (*V*(*T*)) he is expected to mate with at that time and the encounter rate (*φ*). Each mating contributes equally to reproductive success, and pre-emergence mortality is not taken into account (cf. [10]). Pre-emergence mortality has little impact on our results because it would only shape the emergence curve to be slightly left-skewed.

Thus, the expected number of matings achieved by males emerging on average *τ* time units before the females is given by

$$\begin{array}{c} E\left(\tau \right)=\int_{-\infty }^{\infty }\Phi \left(T\right)\frac{e^{T+\tau }}{{\left(1+e^{T+\tau }\right)}^{2}}dT \end{array}$$ (7)

In equation (7), male emergence is described through the (scaled) logistic probability function (see (5)). For numerical optimisation we use a transformed version (for formal proof see S2 appendix), which simplifies the implementation of the model:

$$\begin{array}{c} E\left(\tau \right)=\int_{-\infty }^{\infty }\frac{\varphi }{\psi }M\left(T,\tau \right)V\left(T,\tau \right)dT \end{array}$$ (8)

From the females’ perspective, the optimal sSDT (*τ*) is achieved when *τ* maximises the total number of matings

$$\begin{array}{c} \max(E(\tau ))\;\text{with}\;\tau \in R\text{.} \end{array}$$ (9)

As the moment of fertilisation is not relevant for a female’s reproductive success this minimises the risk to die as a virgin. Due to the lack of any frequency-dependence on the females’ side this is the female ESS.

It is not assured, however, that the sSDT (*τ*) maximising the total number of matings is also a male ESS because mutant males hatching with a different mean time of eclosion might gain above average copulations. To find the corresponding sSDT (*τ*) for the male ESS, we thus assume that rare mutant males (cf. [10] page 312f]) with a different mean time of eclosion $\left({\bar{\mu }}_{m}\right)$ do not have an impact on the scaled density of virgin females in the resident population. The expected number of matings of mutant males thus depends on the sSDT (*τ*) of non-mutant (resident) males and on the sSDT ($\bar{\tau }$) of the mutant males. Under this assumption the expected number of matings for a mutant male is

$$\begin{array}{c} \bar{E}\left(\bar{\tau }\right)=\int_{-\infty }^{\infty }\frac{\varphi }{\psi }M\left(T,\bar{\tau }\right)V\left(T,\tau \right)dT \end{array}$$ (10)

According to the ‘adaptive dynamics approach’ (see [32]) the solution for the male ESS is the point where sSDT ($\bar{\tau }$) of the mutant males—which solves $\max(E\bar{\Phi }(\bar{\tau }))$—is equal to the sSDT (*τ*) of the resident males (see [10]). If a strategy is ESS, convergence-stable stabilising selection will promote the evolution of a monomorphic trait distribution [32]. A joint ESS occurs under conditions where the male and female ESS coincide.

Integrals were numerically approximated by the trapezoidal rule with equally spaced panels and optimisation was based on the functions “optimize” and “uniroot” of the statistics package R [33].

We are especially interested whether the existence of different mortality rates for the two sexes and/or different sex-ratios (at birth, respectively eclosure) may promote the evolution of protogyny, i.e., negative sSDT (*τ*) values. For this purpose we determined the female and male ESS for two different scaled encounter rates (*φ* ∈ {2, 30}). Different combinations of scaled sex specific mortality rates (*λ* ∈ [1/10; 10]) are equally spaced on the log scale such that *λ* = 1.258925^*k*^ | *k* ∈ {−10, − 9, …, 10} and different sex ratios (*ψ* ∈ {0.1, 0.5, 0.9}).

## Results

For all tested parameter combinations we find a unique solution for the level of sSDT (*τ*) of resident males which solves Eq. (10) so that no mutant male with a different level of sSDT ($\bar{\tau }$) can expect to achieve more matings. We verified that all solutions are ESS-stable and convergence-stable according to the criteria provided by Geritz et al. [32]. In the case of females, the existence of an ESS is trivial as—according to model assumptions—females do not compete over access to mating partners.

**Fig 1:**
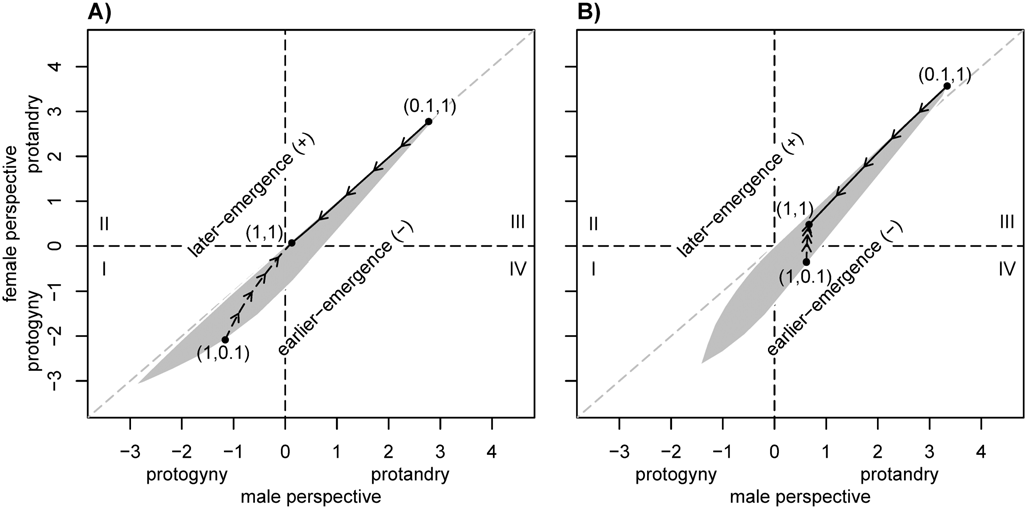
Pairwise plots of male and female evolutionary stable (ESS) SDT. Values on the bisector represent equal male and female ESS. (a) Results for low encounter rate *φ* = 2 and (b) for high encounter rate *φ* = 30; fraction of males is *ψ* = 0.5. The arrows indicate direction of change in pairwise ESS as male mortality rate increases from 0.1 to 1 with female mortality fixed at 1.0. The short-dashed arrows indicate the change in pairwise ESS as female mortality increases from 0.1 to 1 whereas male mortality is fixed at 1.0. The underlying shaded area shows location of pairwise ESS for all combinations of female and male mortalities tested with *λ*_*m*_ and *λ*_*f*_ ∈ {0.1, 0.16, 0.25, 0.4, 0.63, 1}

**Fig 2:**
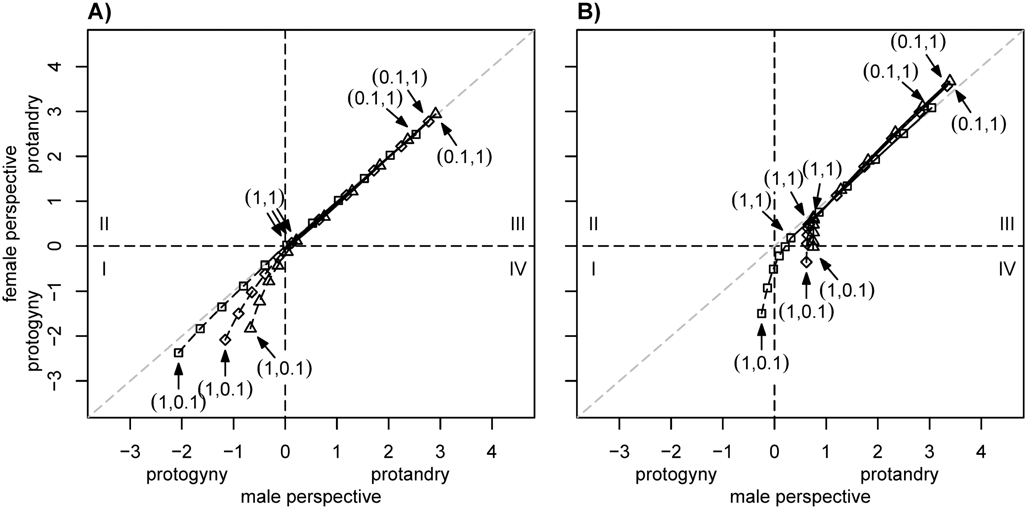
Effect of variation in the sex-ratio (proportion of males *ψ* at moment of eclosure) on pairwise ESS of SDT. Values on the bisector represent equal male and female ESS. (a) Results for low encounter rate *φ* = 2 and (b) for high encounter rate *φ* = 30. Squares—*ψ* = 0.1, diamonds—*ψ* = 0.5 (as in fig. 1), triangles—*ψ* = 0.9. Other parameters as in fig. 1.

Protandry is the only strategy for males and females if the mortality rates are equal as was previously shown by Zonneveld and Metz [10]. The differences between male and female ESS are low, as indicated by the fact that the ESS for the two genders tends to fall onto the main diagonal in figs. 1 and 2. The optimal level of protandry depends on mortality rates: Increasing mortality results in a decline of the optimal time gap in eclosure between males and females; this has also been shown by Zonneveld and Metz [10].

With the introduction of unequal mortality rates protandry is not necessarily the optimal strategy anymore: Especially in scenarios with low encounter rates (*φ* = 2) and where females have considerably lower mortality rates than males, protogyny can become an optimal strategy from the perspective of both sexes (fig. 1A). In addition, with unequal mortality, the male ESS may substantially deviate from the female ESS (see shaded areas in fig. 1A and B). Across the range of all combinations for mortality rates analysed, females would generally favour an earlier emergence than is in the interest of males (the shaded area in figures nearly completely falls below the main diagonal). Indeed, for some combinations of mortality rates (low female and intermediate male mortalities), females may favour protogyny while males would favour protandry (sector IV of figs. 1 and 2). These discrepancies in female and male ESS imply a potential for an evolutionary conflict or arms race, a point we will return to in the discussion.

An increase in encounter rate (*φ*) shifts the general range of resulting ESS towards protandry, especially from the males’ perspective (fig. 1B). The evolution of protogyny may become less likely under these conditions, but the potential for an evolutionary conflict also becomes even more pronounced. Under conditions of very low mortality of females and very high male mortality the former would ‘prefer’ considerable protogyny while the latter would prefer eclosure of females and males at about the same time. With a high encounter rate (*φ* = 30), we also find mortality combinations where females would prefer to emerge slightly later compared to the interest of males; this only occurs under the assumption of low male mortality (see sector III in fig. 1B).

A modification of the sex-ratio (at eclosure) also has an effect on the optimal ESS of both sexes (fig. 2). A reduction in the proportion of males (*ψ*) below 0.5 (at birth, respectively eclosure) tends to favour the evolution of protogyny, while an increase in this proportion shifts the spectrum of ESS towards protandry. Provided that males are rare in the population, protogyny may also become a male ESS, but this does not occur for an even sex-ratio (fig. 2B).

In summary, across all scenarios tested, increasing male mortality in relation to that of females shifts the ESS for both sexes in the direction of protogyny, while the inverse (shift towards protandry) results if we assume high female mortality compared to that of males. High mortality rates of both sexes can only lead to a weak level of SDT of any kind.

## Discussion

Our model—that introduces a straightforward generalisation of the model presented by Zonneveld and Metz [10]—provides the first explanation for the evolution of protogyny as a fitness maximizing strategy in Lepidoptera. Additionally, we show for the first time that—under certain conditions—a pronounced sexual conflict about the optimal SDT can emerge. We found that protogyny is a favourable strategy from the female perspective when male survival is (considerably) lower than that of females (fig. 1 and 2). Intuitively, this makes sense, as long-lived females do not risk much—in terms of survival—when emerging ahead of males but clearly ensure maximal mating chance. More precisely, the prerequisites for protogyny to be an ESS from the females’ perspective are: (i) Males are capable of multiple mating; (ii) females mate only once; and (iii) female longevity is higher compared to that of males. To make protogyny an ESS for males, the encounter rate between males and females must be low.

Bagworms are an example where these critical assumptions are met: Males are capable of multiple mating, females mate only once, and life expectancy of females is much longer than that of males [19]; such extreme differences in longevity of genders are also known from species belonging to the Diptera order [27, 28]. In bagworms the extreme discrepancy in survival may emerge because males carry the cost and risk of searching for receptive females, while females attract males by using pheromones [19]. However, for protogyny to be an adaptive male strategy, a low encounter rate is a further condition. There is indeed evidence for low encounter rates in bagworms: (1) High female mating failures are reported (up to 30%), (2) parthenogenesis, which is rare in Lepidoptera, evolved independently in many genera [1, 19], and (3) aptery, which occurs in over half of the known bagworm species and in 9 out of 10 subfamilies, presumably reduces encounter rates [24, 34].

Alternative explations for the emergence of protogyny exists; Kokko et al. [35] provided an explanation for protogyny in territorial birds, showing that competition can promote the evolution of earlier female arrival to breading areas. However, Kokko et al. only predict evolution of protogyny for low levels of mate competition and female-biased sex ratios (high level of concurrence); bagworms most likely do not meet these restrictions [19]. If we take single mating (Kokko [35]) and multiple mating (with no cost, assumption of our model) as extremes, it is likely that, for anything in between, protogyny can also be an adaptive strategy. A possible non-adaptive explanation for the emergence of SDT is sexual size dimorphism. Such dimorphism is common in butterflies and considered to be promoted by gender specific selection pressure on body size. In a recent study, Teder [36] found a strong correlation between size dimorphism and sexual differences in larval development time. He concluded that attaining a larger body size is not feasible without prolonged larval development. Accordingly, protandry emerges due to selection for large females body size that correlates positively with fecundity. Indeed is such correlation especially strong in capital breeders, a life-history attribute ubiquitous in bagworms [34, 37, 38]. Yet obviously this reasoning provides an argument for the evolution of protandry whereas protogyny is the norm in bagworms.

We found that for a given set of parameters and equal mortality rates, the ESS of the two sexes are nearly identical and therefore always very close to the main diagonal of the pairwise plots (fig. 1). However, with unequal mortality we observe substantial deviations from the main diagonal in these plots, especially in scenarios where females are long-lived and the encounter rate is large (sector I and IV of fig. 1B). This conflict can indeed be substantial: Assuming that the male ESS would prevail, the predicted fraction of mated females could be up to 5% lower than would be the case under the female ESS. It is noteworthy that strong deviations always fall below the main diagonal; this implies that (long-lived) females would generally have an interest in emerging than is in the males’ interest. Under conditions where protandry evolves (sector III in figs. 1 and 2), females would in most scenarios have an interest in lower SDT than males. In turn, where protogyny is predicted, females should prefer a larger time-gap in emergence than the males. The most striking conflict over SDT emerges when females would prefer protogyny and males protandry (sector IV of figures), e.g., when female survival is higher than that of males and the encounter rate is large. In this case, males would prefer to emerge before females, while females would prefer to emerge before males. The existence of such a discrepancy could lead to an ever ongoing evolutionary race. Our model does not predict the evolutionary outcome of this conflict; however, it does identify that such a conflict exists.

Based on the assumptions we made, it follows directly that if the corresponding SDT of the female and male ESS is not identical, there should be an ongoing evolutionary shift (see Eq. (11)) of mean eclosion time to either earlier or later emergence.

$$\begin{array}{c} \mathrm{sign}\left({\text{ESS}}_{m}-{\text{ESS}}_{f}\right)=\left\{\begin{array}{cc} - & \text{shift}\;\text{to}\;\text{earlier-emergence}\\ + & \text{shift}\;\text{to}\;\text{later-emergence} \end{array}\right. \end{array}$$ (11)

A positive value for the difference emerges wherever points in the pairwise ESS plots fall above the main diagonal, and a negative value emerges where points fall below the diagonal (the more frequent outcome).

In nature, timing of emergence quite certainly underlies several external constraints, e.g., those imposed by adverse climatic conditions or the time-limited availability of critical resources such as egg-laying plants or food sources. Such constraints could ultimately define the outcome of the conflict. For example, if males prefer a larger ‘protandrouse time-gap’ than females, the evolutionary race would drive the system to earlier and earlier emergence dates. In this case it appears likely that the males are ultimately prevented from earlier emergence by harsh climatic conditions, and females may consequently enforce their ESS. However, if, for example, the availability of host plants ‘anchors’ the best time for emergence for females, the level of asynchrony may settle at a value closer to the males’ ESS.

Whether one gender can more directly impose its preferred SDT on the other sex is an interesting question. For example, multiple mating as a female strategy affects this conflict and might allow females to influence the ‘game’ in their interest. In a previous paper, Zonneveld [39] showed for even mortality rates that if females mate several times male eclosure time would be selected to be later than that evolving under the assumption of single mating (resulting in a shift to the left of the male ESS in the figures). Under certain conditions (especially those occurring in sector I and IV of figures), multiple mating could thus reduce, if not resolve, the timing of emergence conflict.

In conclusion, according to our model, protogyny and sexual conflict is a likely evolutionary outcome when a substantial risk of mating failure exists and waiting costs for mates are low for females. High mating failures may occur because males are short-lived, encounter rates are low, or males are rare compared to females, i.e., whenever the operational sex-ratio (M/F) is small. The model predictions seem to be in good agreement with the specific case of the bagworm life history and may also be applicable to other insects and non-territorial animals in general.

## Supporting Information

S1 appendix (PDF)

S2 appendix (PDF)
