# Supplementary material for: High Female Survival Promotes Evolution of Protogyny and Sexual Conflict
Source: PLoS One. 2015 Mar 16;10(3):e0118354. doi: 10.1371/journal.pone.0118354 (PMC4361667; doi:10.1371/journal.pone.0118354)
Supplement: S1 appendix — (PDF) [file pone.0118354.s001.pdf]

Appendix S1

The sollution of equation (3) and (4) with initial conditions $\lim_{T\to-\infty} M(T) = 0$ and $\lim_{T\to-\infty} V(T) = 0$:

$$M(T;\tau;\lambda_m;\psi) = \psi e^{-\lambda_m(T+\tau)} \int_0^{e^{T+\tau}} \frac{x^{\lambda_m}}{(1+x)^2} dx \tag{1}$$

and

$$V(T;\tau;\lambda_m;\lambda_f\psi) = (1-\psi)e^{-(\lambda_f + f(T;\tau;\lambda_m;\psi))} \int_0^{e^T} \frac{x^{\lambda_f} e^{f(\log(x);\tau;\lambda_m;\psi)}}{(1+x)^2} dx \tag{2}$$

with

$$f(y;\tau;\lambda_m;\psi) = \varphi \int_{-\infty}^{y} M(u;\tau;\lambda_m;\psi) du \tag{3}$$
