# Supplementary material for: High Female Survival Promotes Evolution of Protogyny and Sexual Conflict
Source: PLoS One. 2015 Mar 16;10(3):e0118354. doi: 10.1371/journal.pone.0118354 (PMC4361667; doi:10.1371/journal.pone.0118354)
Supplement: S2 appendix — (PDF) [file pone.0118354.s002.pdf]

Appendix S2

The proof of equality of (5) and (6): The expected number of matings for males emerging at scaled time T is

$$\Phi(T) = \varphi e^{\lambda_m T} \int_T^{\infty} e^{-\lambda_m t} V(t) dt$$

The total number of expected matings is

$$E(\tau) = \int_{-\infty}^{\infty} \Phi(T) \frac{e^{T+\tau}}{(1+e^{T+\tau})^2} dT$$

Replacing the fraction in the differential equation and separating the terms yields

$$\begin{aligned} E &= \int_{-\infty}^{\infty} \frac{1}{\psi} \left[\dot{M}(T) + \lambda_m M(T) \right] \Phi(T) dT \\ &= \frac{1}{\psi} \left[\int_{-\infty}^{\infty} \dot{M}(T)\Phi(T) dT + \lambda_m \int_{-\infty}^{\infty} M(T)\Phi(T) \right] \end{aligned}$$

with $\dot{M}(T) = \frac{\partial}{\partial T} M(T)$. Partial Integration of the first term yields

$$= \frac{1}{\psi} \left[\Phi(T)M(T)|_{-\infty}^{\infty} - \int_{-\infty}^{\infty} \dot{\Phi}(T)M(T)dT + \lambda_m \int_{-\infty}^{\infty} M(T)\Phi(T) \right]$$

Obviously the first term is equal 0. Calculation of the derivative in the middle term yields

$$\begin{aligned} \dot{\Phi}(T) &= \varphi \frac{\partial}{\partial T} \left[e^{\lambda_m T} \int_T^{\infty} e^{-\lambda_m t} V(t) dt \right] \\ &= \varphi \left[\lambda_m e^{\lambda_m T} \int_T^{\infty} e^{-\lambda_m t} V(t) dt - e^{\lambda_m T} e^{-\lambda_m T} V(T) \right] \\ &= \lambda_m \Phi(T) - \varphi V(T) \end{aligned}$$

Thus E is

$$\begin{aligned} E &= \frac{1}{\psi} \left[- \int_{-\infty}^{\infty} \left(\lambda_m \Phi(T) - \varphi V(T) \right) M(T) dT + \lambda_m \int_{-\infty}^{\infty} M(T)\Phi(T) dT \right] \\ &= \frac{1}{\psi} \left[-\lambda_m \int_{-\infty}^{\infty} \Phi(T)M(T)dT + \int_{-\infty}^{\infty} \varphi V(T)M(T)dT + \lambda_m \int_{-\infty}^{\infty} M(T)\Phi(T)dT \right] \\ &= \frac{1}{\psi} \int_{-\infty}^{\infty} \varphi V(T) M(T) dT \end{aligned}$$
